# The relative abundance of fecal bacterial species belonging to the *Firmicutes* and *Bacteroidetes* phyla is related to plasma levels of bile acids in young adults

**DOI:** 10.1007/s11306-023-02016-8

**Published:** 2023-06-06

**Authors:** Francisco J. Osuna-Prieto, Huiwen Xu, Lourdes Ortiz-Alvarez, Xinyu Di, Isabelle Kohler, Lucas Jurado-Fasoli, Jose Rubio-Lopez, Julio Plaza-Díaz, Ramiro Vilchez-Vargas, Alexander Link, Angel Gil, Jonatan R. Ruiz, Patrick C. N. Rensen, Borja Martinez-Tellez

**Affiliations:** 1 Department of Physical Education and Sports, Faculty of Sport Sciences, PROmoting FITness and Health through Physical Activity Research Group (PROFITH), Sport and Health University Research Institute (iMUDS), University of Granada, Granada, Spain; 2 Department of Biochemistry and Molecular Biology II, Institute of Nutrition and Food Technology, Center for Biomedical Research, University of Granada, Granada, Spain; 3 Metabolomics and Analytics Centre, Leiden Academic Centre for Drug Research (LACDR), Leiden University, Leiden, The Netherlands; 4 Division of BioAnalytical Chemistry, Amsterdam Institute of Molecular and Life Sciences (AIMMS), Vrije Universiteit Amsterdam, Amsterdam, The Netherlands; 5 Center for Analytical Sciences Amsterdam, Amsterdam, The Netherlands; 6 Department of Physiology. Faculty of Medicine, University of Granada, Av.Conocimiento S/N, 18011 Granada, Spain; 7 Cirugía General Y del Aparato Digestivo, Complejo Hospitalario de Jaen, Jaén, Spain; 8 Children’s Hospital of Eastern Ontario Research Institute, Ottawa, ON K1H 8L1 Canada; 9 Instituto de Investigación Biosanitaria, ibs. Granada, Granada, Spain; 10 Department of Gastroenterology, Hepatology and Infectious Diseases, Otto-von-Guericke-University Magdeburg, Magdeburg, Germany; 11 CIBEROBN, Biomedical Research Networking Center for Physiopathology of Obesity and Nutrition, Carlos III Health Institute, Madrid, Spain; 12 Department of Medicine, Division of Endocrinology, and Einthoven Laboratory for Experimental Vascular Medicine, Leiden University Medical Center, Leiden, The Netherlands; 13 Department of Education, Faculty of Education Sciences and SPORT Research Group (CTS-1024), CERNEP Research Center, University of Almería, Almería, Spain; 14 Department of Analytical Chemistry, University of Granada, 18071 Granada, Spain

**Keywords:** 7-α-Dehydroxylases, Bile salt hydrolases, Gut microbiota, Microbiome

## Abstract

**Background:**

Gut bacteria play a crucial role in the metabolism of bile acids (BA). Whether an association exists between the fecal microbiota composition and circulating BA levels in humans is poorly understood. Here, we investigated the relationship between fecal microbiota diversity and composition with plasma levels of BA in young adults.

**Methods:**

Fecal microbiota diversity/composition was analyzed with 16S rRNA sequencing in 80 young adults (74% women; 21.9 ± 2.2 years old). Plasma levels of BA were measured using liquid chromatography-tandem mass spectrometry. PERMANOVA and Spearman correlation analyses were used to investigate the association between fecal microbiota parameters and plasma levels of BA.

**Results:**

Fecal microbiota beta (P = 0.025) and alpha diversity indexes of *evenness* (rho = 0.237, P = 0.033)*, Shannon* (rho = 0.313, P = 0.004)*,* and *inverse Simpson* (rho = 0.283, P = 0.010) were positively associated with plasma levels of the secondary BA glycolithocholic acid (GLCA). The relative abundance of genera belonging to the *Firmicutes* and *Bacteroidetes* phyla was positively correlated with plasma levels of GLCA (all rho ≥ 0.225, P ≤ 0.049). However, the relative abundance of species from *Firmicutes* and *Bacteroidetes* phyla were negatively correlated with plasma levels of primary and secondary BA (all rho ≤ − 0.220, P ≤ 0.045), except for the relative abundance of *Bacteroides vulgatus, Alistipes onderdonkii,* and *Bacteroides xylanisolvens* species (*Bacteroidetes* phylum) that were positively correlated with the plasma levels of GLCA.

**Conclusions:**

The relative abundance of specific fecal bacteria species is associated with plasma levels of BA in young adults. However, further investigations are required to validate whether the composition of the gut microbiota can regulate the plasma concentrations of BA in humans.

**Supplementary Information:**

The online version contains supplementary material available at 10.1007/s11306-023-02016-8.

## Introduction

The gut microbiota is composed of a complex set of microorganisms that colonize the gastrointestinal tract, where bacteria are the most abundant (Sekirov & Finlay, 2006). The most abundant bacteria present in the human gut microbiota belong to the *Firmicutes* and *Bacteroidetes* phyla (Mariat et al., 2009), representing more than 70% of the total bacteria (Rinninella et al., 2019). Gut microbiota composition is modulated by extrinsic factors, such as diet (David et al., 2014), exercise (O’Sullivan et al., 2015), and medication (Maier et al., 2018; Wu et al., 2017), as well as by intrinsic factors, such as age (Lozupone et al., 2012) and host genetics (Bonder et al., 2016). Recent advances have shown that the metabolites of these bacteria influence several biological processes, such as digestion and absorption of nutrients, as well as the homeostatic maintenance of host immunity and gut barrier permeability (Montalto et al., 2009; van de Guchte et al., 2018). For this reason, the impact of gut microbiota on human metabolism depends not only on their abundance but also on the metabolites that they produce (Gózd-Barszczewska et al., 2017).

The primary bile acids (BA) cholic acid (CA) and chenodeoxycholic acid (CDCA) are synthesized from cholesterol in hepatocytes, where they are typically conjugated with glycine to produce glycocholic acid (GCA) and glycochenodeoxycholic acid (GCDCA), respectively, as well as taurine to produce the corresponding tauro-conjugates (Ridlon et al., 2016); glycine-conjugated BA represent ~ 75% of the total pool of conjugated BA (Wahlström et al., 2016). Primary BA are then stored in the gallbladder and released into the duodenum to facilitate the absorption of dietary lipids and liposoluble vitamins, protect against bacterial overgrowth (Begley et al., 2005), and eliminate excess cholesterol (Suga et al., 2019; Wahlström et al., 2016). Approximately 95% of the BA are reabsorbed within the distal ileum and returned to the liver through the enterohepatic circulation (Zwicker & Agellon, 2013). The remaining 5% of primary BA enter the colon, where they are metabolized into the secondary BA deoxycholic acid (DCA), lithocholic acid (LCA), and ursodeoxycholic acid (UDCA) upon the action of specific bacterial enzymes (Ridlon et al., 2006).

Bile salt hydrolases (BSHs) and 7-α-dehydroxylases are the major bacterial enzymes involved in the metabolism of BA (De Smet et al., 1995). BSHs participate in the deconjugation of primary and secondary BA by catalyzing the removal of glycine and taurine (De Smet et al., 1995); these enzymes are present in certain Gram-positive bacteria of the phylum *Firmicutes* and Gram-negative bacteria of the phylum *Bacteroidetes* (Jones et al., 2008; Long et al., 2017; Mullish et al., 2018). The 7-α-dehydroxylases enzymes convert primary BA to secondary BA and are expressed by certain anaerobic bacteria, such as Gram-positive bacteria species belonging to the *Clostridium* genus (Long et al., 2017; Mullish et al., 2018). These secondary BA can either be reabsorbed by colonocytes (Krag & Phillips, 1974), pass to systemic circulation inducing signaling functions (Hirokane et al., 2004; Stayrook et al., 2005), and reach the liver via the enterohepatic circulation where they can again be conjugated with glycine or taurine (Hofmann, 1999), or be secreted into the feces (Begley et al., 2005). However, it is unknown whether a relationship exists between bacterial species expressing BSHs and 7-α-dehydroxylases and circulating levels of primary and secondary BA in humans. Based on that, we hypothesize that certain bacterial communities within the human gut are related to plasma levels of primary and secondary BA.

The aim of the present study was to investigate the relationship of fecal microbiota diversity and composition with plasma levels of primary and secondary BA in a cohort of young adults.

## Material & methods

### Participants

This study was conducted using baseline data from the ACTIBATE project (Activating brown adipose tissue through exercise in young adults) (Sanchez-Delgado et al., 2015), a randomized controlled trial designed to evaluate the effect of exercise training on brown adipose tissue (BAT) activity (Clinical trials identifier: NCT02365129). The University of Granada recruited participants via advertisements in electronic media and leaflets. The inclusion criteria were: being sedentary, i.e., less than 20 min of moderate/vigorous physical activity on < 3 days/week and have had a stable body weight over the last 3 months. The exclusion criteria were: being pregnant, smoker, being frequently exposed to cold temperatures, presenting any acute or chronic disease (e.g., hypertension or diabetes) that can interfere with or be aggravated by exercise, taking medication that potentially affects the cardiovascular system and/or its function in the last 3 months. The study protocol and the written informed consent were performed in accordance with the last revised Declaration of Helsinki and were approved by the Ethics Committee on Human Research of the University of Granada (nº.924), and Servicio Andaluz de Salud (Centro de Granada, CEI-Granada).

We selected participants from the ACTIBATE study with available data for fecal microbiota and plasma samples, which resulted in 80 young adults (59 women, 21 men, aged 18–25 years) included in the present study. All the research visits took place at the Instituto Mixto Universitario Deporte y Salud (iMUDS) center in Granada (Spain).

### Fecal microbiota analysis

A fecal sample (50–60 g) was obtained from each volunteer and introduced in a 60 mL plastic sterile container. The fecal samples were transported in a portable cooler with an ice plate to the research center and stored at – 80 °C until DNA extraction. Fecal samples were homogenized in a Stomacher® 400 blender (A. J. Seward and Co. Ltd., London, UK). DNA extraction and purification steps were performed with a QIAamp DNA Stool Mini Kit (QIAGEN, Barcelona, Spain) according to the manufacturer’s instructions. DNA concentration and purity were determined with a NanoDrop ND1000 spectrophotometer (Thermo Fisher Scientific, DE, USA).

DNA was amplified by PCR in 16S targeting the V3 and V4 hypervariable regions. The amplicons were sequenced in a MiSeq (Illumina, San Diego, CA, USA), using the Illumina MiSeq paired-end sequencing system (2 × 300nt) (Illumina, San Diego, CA, USA). We used the “*dada2*” package version 1.10.1 in *R* software (R Core Team, 2019) was used for merging and filtering raw sequences (FastQ files). Ribosomal Data Project (RDP) (Cole et al., 2014) was used to assign the phylotypes to their specific taxonomic affiliation (from phylum to genus). The methodology is described in detail in the Supplementary Material.

### Determination of plasma levels of bile acids

Blood samples were collected between 8:00 and 9:00 AM after 10-h overnight fasting. Blood samples were collected in Vacutainer Tubes® and immediately centrifuged. Serum (obtained with Vacutainer® SST™ II Advance tubes) and plasma (obtained with Vacutainer® Hemogard™ tubes) aliquots were stored at − 80 °C until analyses. Primary (i.e., CA, CDCA, GCA, and GCDCA,) and secondary BA (i.e., DCA, glycodeoxycholic [GDCA], glycolithocholic [GLCA], glycoursodeoxycholic [GUDCA]) were measured in plasma samples, using liquid chromatography-tandem mass spectrometry (LC–MS/MS), using a method validated according to the FDA bioanalytical method validation guidelines (FDA, F. and D. A. 2018). The methodology is described in detail in the Supplementary material.

### Anthropometry and body composition

A SECA model 799 electronic column scale and stadiometer (SECA, Hamburg, Germany) were used to measure participants´ height and weight, without shoes and wearing standard clothes. A dual-energy X-ray absorptiometry scan (Hologic Discovery Wi Marlborough, MA) was used to measure body composition (fat mass and lean mass). The body mass index (BMI) was calculated as weight/height^2^ (kg/m^2^).

### Cardiometabolic parameters profile

Fasting serum glucose, total cholesterol (TC), high-density lipoprotein cholesterol (HDL-C), and triacylglycerols concentrations, in mg/dL, were measured following standard methods using an AU5832 biochemical analyzer (Beckman Coulter Inc., Brea, CA, USA) with Beckman Coulter reagents OSR6521, OSR6116, OSR60118, and OSR6187, respectively. Low-density lipoprotein cholesterol (LDL-C) was estimated with the Friedewald formula: [TC—HDL-C—(TG/5)], in mg/dL (Friedewald et al., 1972). Insulin was measured using the Access Ultrasensitive Insulin Chemiluminescent Immunoassay Kit (Beckman Coulter Inc., Brea CA, USA) in µUl/mL. The homeostatic model assessment (HOMA) index was calculated as [insulin (µU/mL) × glucose (mmol/L)/22.5] (Matthews et al., 1985).

### Dietary assessment

The dietary assessment has been explained in detail elsewhere (Jurado-Fasoli et al., 2020). We assessed the dietary intake (energy and nutrient intake) from three 24-h dietary recalls. The 24-h dietary recalls were undertaken on 3 separate days (2 weekdays, and one weekend day) with face-to-face interviews by qualified and trained dietitians. Two dieticians introduced all data from interviews independently into EvalFINUT® software (https://www.finut.org/evalfinut/).

### Statistical analysis

The descriptive parameters are reported as mean and standard deviation. Since we did not observe sex interaction in our cohort (all P ≥ 0.05), men and women were analyzed together. D’Agostino & Pearson tests revealed that the relative abundance of the different bacterial species within the feces, plasma levels of BA, and serum cardiometabolic profile parameters followed a non-normal distribution. Therefore, all the analyses were conducted using non-parametric tests.

In order to investigate the association between fecal microbiota beta diversity and plasma levels of primary and secondary BA, we divided into tertiles of the plasma levels of BA (i.e., low, intermediate, and high concentration), and compared across tertiles using a PERMANOVA analysis with 9999 random permutations, based on Bray–Curtis dissimilarity. This analysis was performed with the Paleontological Statistics Software Package 3.0 (Past3) (Hammer et al., 2001). Four different alpha diversity indexes were calculated using the “*vegan*” package in *R* software. To investigate the association between alpha diversity indexes and plasma levels of primary and secondary BA, we employed Spearman correlation analyses using “p*sych*” and “c*orrplot*” packages in *R* software. Then, to investigate the association between fecal microbiota composition and plasma levels of primary and secondary BA, we used Spearman correlation and partial Spearman correlations analysis using “*psych*” and “c*orrplot*” packages in *R* software. Lastly, to investigate the association between specific bacterial and BA pathways, we used Spearman correlation analysis using “*psych*” and “c*orrplot*” packages in *R* software.

The correlations between alpha diversities indexes and plasma levels of primary and secondary BA were represented as heatmap plots using “g*plot*” package in *R* software. Volcanos plots were used to depict the correlations between fecal microbiota composition (at genus and species taxonomy levels) and plasma levels of primary and secondary BA by using GraphPad Prism software (GraphPad Software, San Diego, California, USA, version 8.0.0). Also, the correlation between specific bacterial species and BA pathways were represented as heatmap plots using GraphPad Prism software (GraphPad Software, San Diego, California, USA, version 8.0.0). The level of significance was set at P < 0.05.

## Results

### Characteristics of participants

Table 1 shows the descriptive characteristics of the participants (74% women; 21.9 ± 2.2 years-old; BMI: 24.7 ± 4.7 kg/m^2^).

**Table 1:** Descriptive characteristics of study participants

		N	Mean ± SD		
Age (years-old)		80	21.9 ± 2.2		
Sex (women, %)		80	73.7% (n = 59)		
Body composition parameters					
Fat mass (kg)		80	25.2 ± 9.1		
Lean mass (kg)		80	41.0 ± 8.9		
Body mass index (kg/m^2^)		80	24.7 ± 4.7		
Cardiometabolic profile parameters					
Glucose (mg/dL)		79	87.6 ± 6.1		
Insulin (µUl/mL)		79	8.1 ± 4.8		
HOMA index		79	1.8 ± 1.2		
Total cholesterol (mg/dL)		79	167.8 ± 36.0		
Total triglycerides (mg/dL)		79	85.1 ± 50.5		
HDL-C (mg/dL)		79	53.5 ± 11.7		
LDL-C (mg/dL)		79	97.0 ± 27.0		
Energy and macronutrient intake					
Energy (kcal/day)		80	1919.4 ± 489.2		
Carbohydrate (g/day)		80	204.9 ± 63.5		
Protein (g/day)		80	76.6 ± 22.0		
Lipids (g/day)		80	86.0 ± 11.7		
Plasma levels of bile acids (expressed as peak area ratio)					
Primary bile acids					
CA		80	28.6 ± 48.5		
CDCA		80	0.5 ± 0.7		
GCA		80	1.9 ± 1.7		
GCDCA		78	5.4 ± 3.6		
Secondary bile acids					
DCA		80	17.3 ± 13.8		
GDCA		79	1.9 ± 1.5		
GLCA		80	4.5 ± 4.1		
GUDCA		80	16.9 ± 18.2		
Fecal microbiota parameters					
Diversity indexes					
Species Richness		80	380.0 ± 109.6		
Evenness Index		80	0.7 ± 0.0		
Shannon Index		80	4.2 ± 0.4		
Inverse Simpson Index		80	35.6 ± 14.5		
Composition (phylum)					
Actinobacteria (%)		80	1.6 ± 1.6		
Bacteroidetes (%)		80	39.6 ± 9.0		
Firmicutes (%)		80	48.8 ± 9.7		
Proteobacteria (%)		80	6.5 ± 4.8		
Verrucomicrobia (%)		80	2.3 ± 4.3		

Data are presented as mean and standard deviation (SD), except for sex. *CA* cholic acid, *CDCA* chenodeoxycholic acid, *DCA* deoxycholic acid, *GCA* glycocholic acid, *GCDC* glycochenodeoxycholic acid, *GDCA* glycodeoxycholic acid, *GLCA* glycolithocholic acid, *GUDCA* glycoursodeoxycholic acid, *HDL*C high-density lipoprotein cholesterol, *LDL-C* low-density lipoprotein cholesterol

### Fecal microbiota diversity positively correlates with plasma levels of glycolithocholic acid

**Fig. 1:**
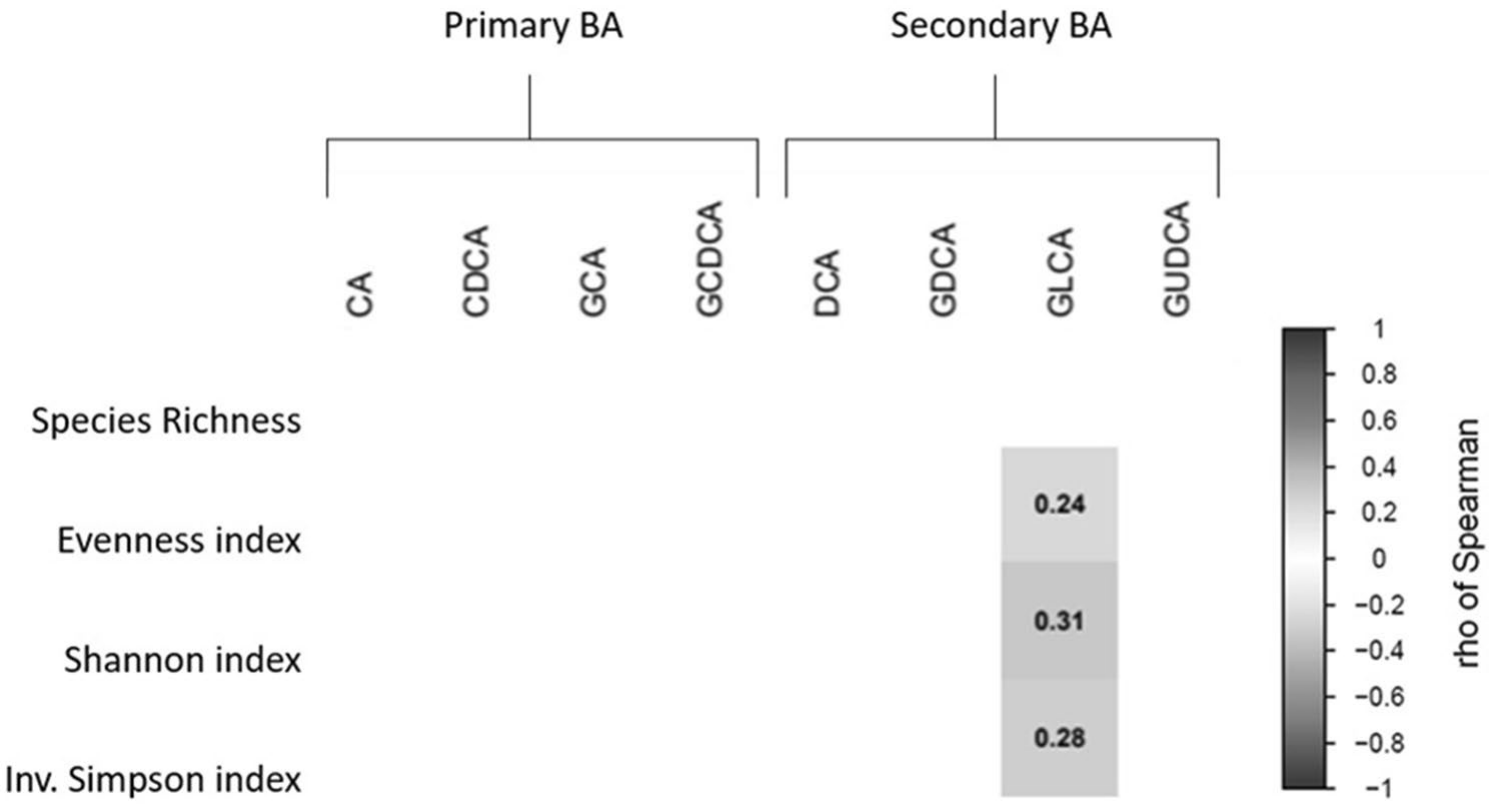
Spearman correlations between fecal microbiota alpha diversity indexes and plasma level of bile acids. Blue boxes represent positive and significant correlations (P < 0.05), while the value within the boxes shows Spearman’s correlations coefficients. BA: Bile acids; CA: cholic acid; *CDCA* chenodeoxycholic acid; *DCA* deoxycholic acid; *GCA* glycocholic acid; *GCDC* glycochenodeoxycholic acid; *GDCA* glycodeoxycholic acid; *GLCA* glycolithocholic acid; *GUDCA* glycoursodeoxycholic acid; Inv: Inverse

We found differences in fecal microbiota beta diversity at genus taxonomy levels only among tertiles (low *vs* high) of plasma levels of the secondary BA GLCA (pseudo-F = 1.845, P = 0.025; Table 2). Additionally, we observed positive correlations of *evenness* (rho = 0.237, P = 0.033)*, Shannon* (rho = 0.313, P = 0.004)*,* and *inverse Simpson* alpha diversity indexes (rho = 0.283, P = 0.010) with plasma levels of GLCA (Fig. 1). No relationships were observed between fecal microbiota beta and alpha diversities and plasma levels of other BA (all P > 0.05).

**Table 2:** Differences in beta diversity calculated by statistic permutational multivariate analysis of variance (PERMANOVA) among tertiles of plasma levels of bile acids (peak area ratio) at phylum and genus taxonomic levels

				Phylum		Genus	
	Low	Medium	High	*Pseudo-F*	*P-value*	*Pseudo-F*	*P-value*
Primary bile acids							
CA	1.874	9.437	73.547	0.411	0.838	1.541	0.072
CDCA	0.093	0.326	1.193	0.510	0.767	1.119	0.305
GCA	0.484	1.349	3.681	1.010	0.401	0.800	0.690
GCDCA	1.802	4.957	9.573	0.541	0.762	0.736	0.820
Secondary bile acids							
DCA	5.465	14.237	31.849	1.892	0.102	1.190	0.250
GDCA	0.521	1.470	3.777	0.676	0.618	1.037	0.381
GLCA	1.419	3.249	8.752	0.237	0.937	1.845	**0.025**
GUDCA	3.810	11.784	34.745	0.607	0.685	0.714	0.806

PERMANOVA using 9999 permutations for significance testing (P < 0.05). *CA* cholic acid, *CDCA* chenodeoxycholic acid, DCA deoxycholic acid, *GCA* glycocholic acid, *GCDC* glycochenodeoxycholic acid, *GDCA* glycodeoxycholic acid, *GLCA* glycolithocholic acid, *GUDCA* glycoursodeoxycholic acid

### The relative abundance of genera belonging to the *Firmicutes* and *Bacteroidetes* phyla is positively correlated with plasma levels of glycolithocholic acid

**Fig. 2:**
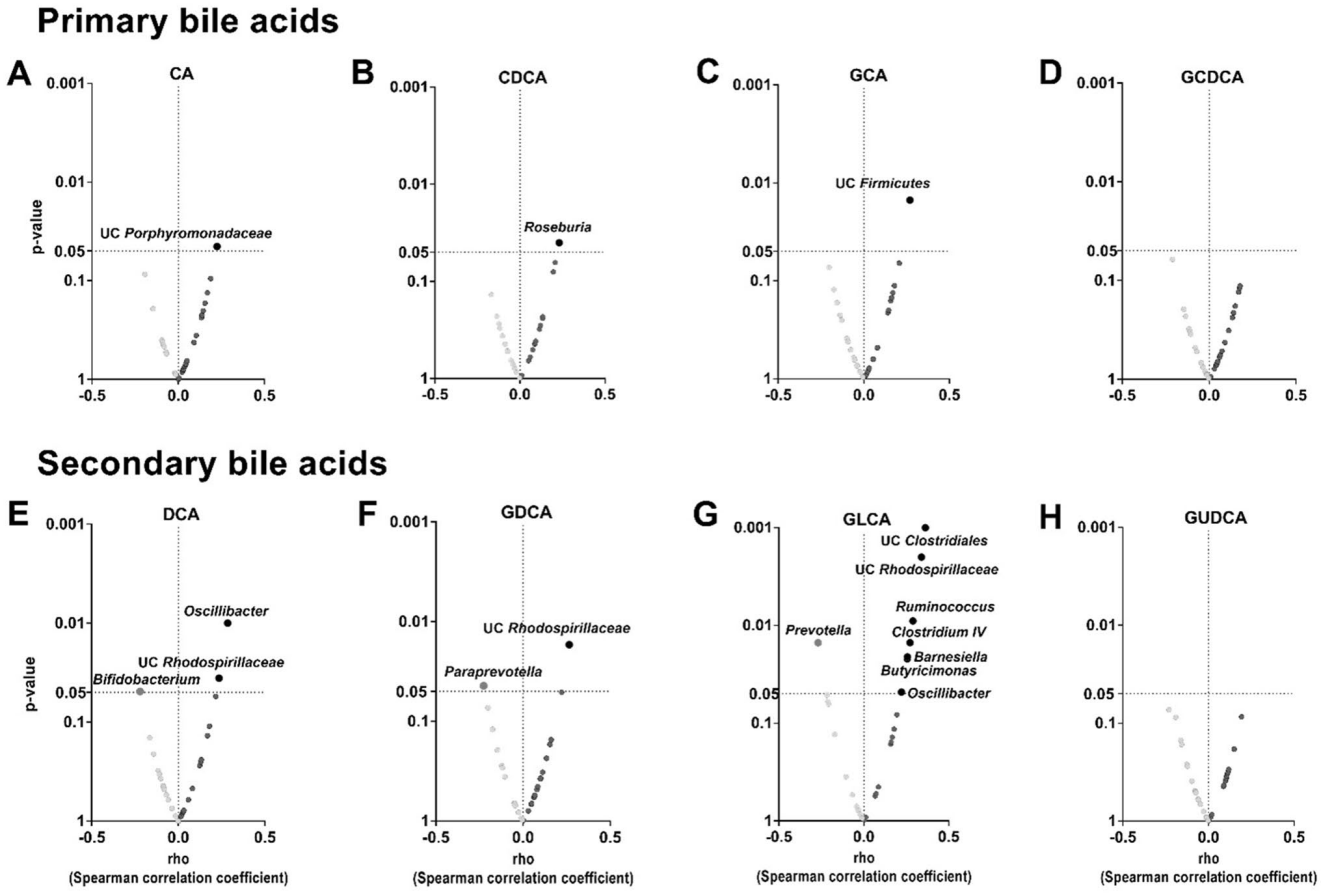
Volcano plots showing Spearman correlations between fecal microbiota composition at genus taxonomic level and plasma level of bile acids. The X-axis represents Spearman’s correlations coefficients, whereas Y-axis represents the P-value of the correlations. Red circles indicate negative correlations, whereas blue circles indicate positive correlations. Only those correlations that achieved the significant threshold (P < 0.05) were annotated with the name of the genera. *CA* cholic acid; *CDCA* chenodeoxycholic acid; DCA: deoxycholic acid; *GCA* glycocholic acid; *GCDC* glycochenodeoxycholic acid; *GDCA* glycodeoxycholic acid; *GLCA* glycolithocholic acid; *GUDCA* glycoursodeoxycholic acid; *UC* unclassified

Overall, we found that the relative abundance of the genera belonging to the different phyla found in the feces was not related to the plasma levels of the primary BA CA (Fig. 2A), GCA (Fig. 2C), and GCDCA (Fig. 2D). Nevertheless, we observed a positive correlation between the relative abundance of the *Roseburia* genus (*Firmicutes* phylum) and the plasma levels of CDCA (rho = 0.231, P = 0.038; Fig. 2B)**.** Furthermore, a positive correlation between the relative abundance of *Oscillibacter* genus (*Firmicutes* phylum) and the plasma levels of the secondary BA DCA and GLCA (both rho ≥ 0.221, P ≤ 0.049; Fig. 2E, G) was found. The relative abundance of the *Unclassified Rhodospirillaceae* genus (*Proteobacteria* phylum) was positively correlated with the plasma levels of DCA, GDCA, and GLCA (all rho ≥ 0.236, P ≤ 0.035; Fig. 2E–G, respectively). We also found that the relative abundance of *Unclassified Clostridiales*, *Unclassified Firmicutes*, *Ruminococcus,* and *Clostridium IV* genera (*Firmicutes* phylum) were positively correlated with plasma levels of GLCA (all rho ≥ 0.263, P ≤ 0.018; Fig. 2G). Additionally, the relative abundance of *Barnesiella* and *Butyricimonas* genera (*Bacteroidetes* phylum) was positively correlated with plasma levels of GLCA (all rho ≥ 0.257, P ≤ 0.023; Fig. 2G). The relative abundance of the *Bifidobacterium* genus (*Actinobacteria* phylum) was negatively correlated with plasma levels of DCA (rho = − 0.047, P = 0.048; Fig. 2E). Finally, the relative abundance of *Paraprevotella* and *Prevotella* genera (*Bacteroidetes* phylum) was negatively correlated with plasma levels of GDCA and GLCA (all rho ≥ -0.270, P ≤ 0.045; Fig. 2F, G). These analyses were repeated after adjusting for BMI, glucose, insulin, HOMA index, and TC, TG, HLD-C, and LDL-C serum levels, and the results remained significant (data not shown).

### The relative abundance of species from *Firmicutes* and *Bacteroidetes* phyla is negatively correlated with plasma levels of primary and secondary bile acids

**Fig. 3:**
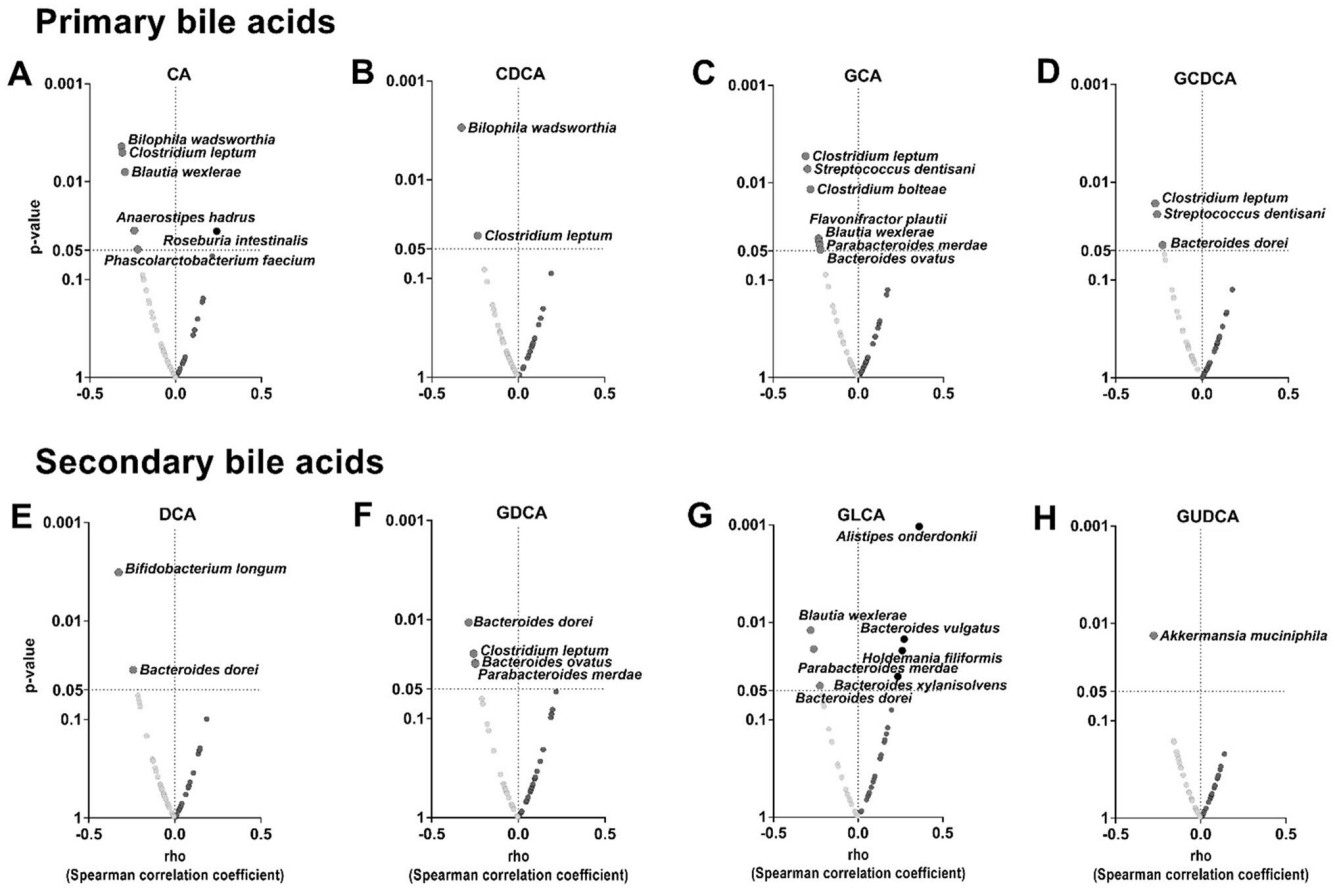
Volcano plots showing Spearman correlation between fecal microbiota composition at species taxonomic level and plasma levels of bile acids. The X-axis represents Spearman’s correlations coefficients, whereas the Y-axis represents the P-value of the correlations. Red circles indicate negative correlations, whereas blue circles indicate positive correlations. Only those correlations that achieved the significant threshold (P < 0.05) were annotated with the name of the species. *CA* cholic acid; *CDCA* chenodeoxycholic acid; *DCA* deoxycholic acid; *GCA* glycocholic acid; *GCDC* glycochenodeoxycholic acid; *GDCA* glycodeoxycholic acid; *GLCA* glycolithocholic acid; *GUDCA* glycoursodeoxycholic acid

We further investigated whether the relative abundance of specific bacterial species could be driving the significant correlations found in the previous analyses (Fig. 2). The relative abundance of C*lostridium bolteae, Clostridium leptum,* and *Blautia wexlerae* species (*Firmicutes* phylum) was negatively correlated with plasma levels of various primary BA (CA, CDCA, GCA, and/or GCDA; all rho ≤ − 0.234, P ≤ 0.039; Fig. 3A–D). Similarly, the relative abundance of *Bacteroides ovatus, Parabacteroides merdae,* and *Bacteroides dorei* species (*Bacteroidetes* phylum) was negatively correlated with the plasma levels of various secondary BA (DCA, GDCA, and GLCA; all rho ≤ − 0.248, P ≤ 0.043; Fig. 3E–G). The relative abundance of *Clostridium leptum* genus was negatively correlated with the plasma levels of all primary BA CA, CDCA, GCA, and GCDCA (all rho ≤ − 0.234, P ≤ 0.038; Fig. 3A–D), as well as the secondary BA GDCA (Rho ≤ − 0.257, P ≤ 0.022; Fig. 3F). Moreover, the relative abundance of *Bacteroides dorei* genus was negatively correlated with the plasma levels of the primary BA GCDCA (Rho = − 0.22, P = 0.043; Fig. 3D), as well as the secondary BA DCA, GDCA, and GLCA (All rho ≤ − 0.22, P ≤ 0.045; Fig. 3E–G). In contrast, the relative abundance of *Bacteroides vulgatus, Alistipes onderdonkii,* and *Bacteroides xylanisolvens* species (*Bacteroidetes* phylum) were positively correlated with the plasma levels of GLCA (all rho ≥ 0.235, P ≤ 0.036; Fig. 3G). These analyses were repeated after adjusting for sex, energy, and macronutrient intake and the results remained unaltered (data not shown).

**Fig. 4:**
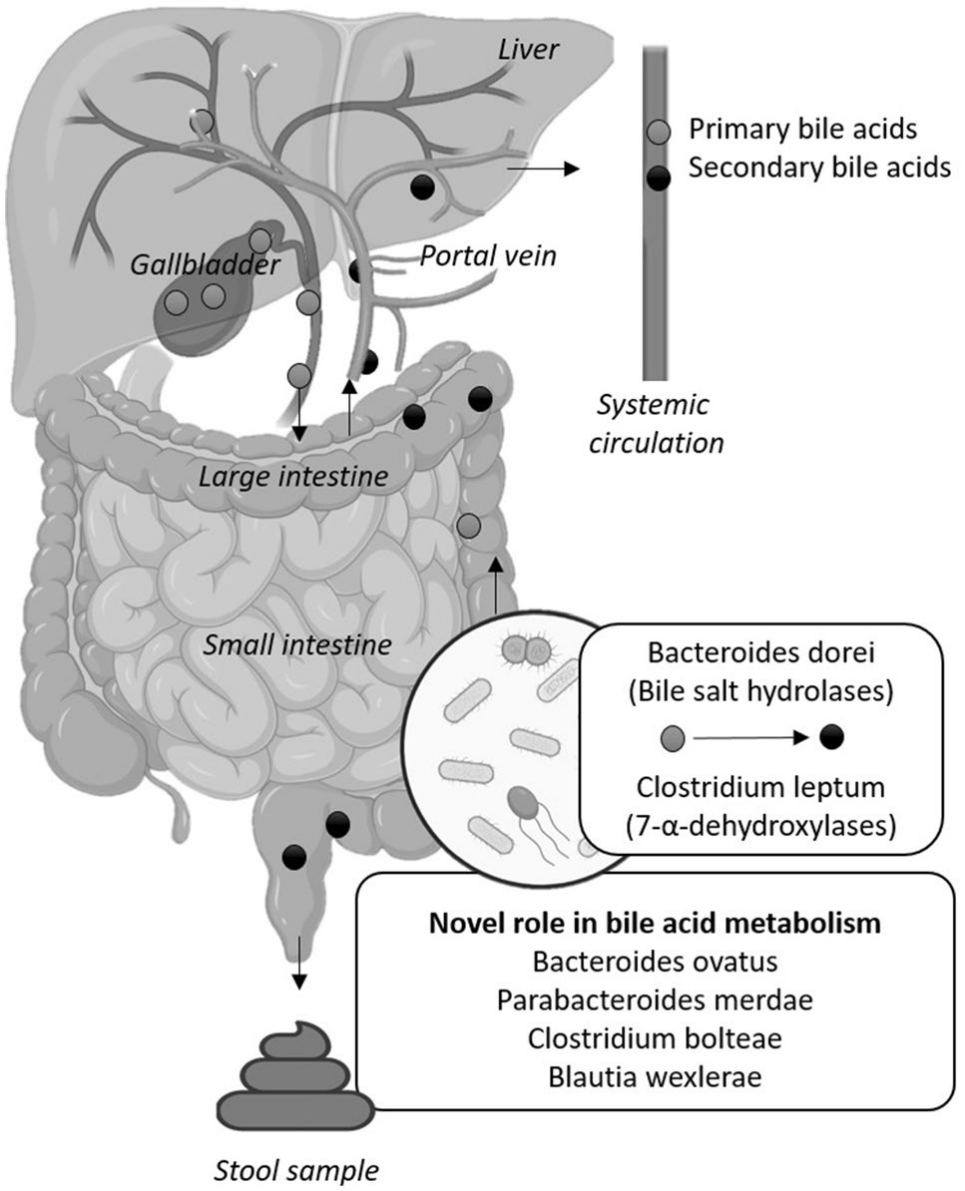
The relative abundance of fecal bacterial species belonging to the *Firmicutes* and *Bacteroidetes* phyla*,* which are known to express bile salt hydrolases (BSHs) and 7-α-dehydroxylases enzymes involved in the metabolism of bile acids, is related to plasma levels of bile acids in young adults. The relative abundance of *Bacteroides ovatus, Parabacteroides merdae, Clostridium boltaeae*, and *Blautia wexlerae* is also related to plasma levels of bile acids, suggesting that they could be directly or indirectly involved in bile acid metabolism in humans. Created with BioRender.com

We predicted bacterial pathways that seem to be involved in BA biosynthesis pathways. Based on that, we found that the relative abundance of *Bacteroides xylanisolvens*, *Bacteroides vulgatus*, *Bifidobacterium longum*, *Bacteroides ovatus,* and *Holdemania filifomis were* positively correlated to the predicted primary and secondary BA biosynthesis pathways (all rho ≥ 0.23, P ≤ 0.039, Figure S1); whereas the relative abundance of *Akkermansia mucinphila* was negatively correlated with the predicted BA biosynthesis pathways (all rho ≤ − 0.27, P ≤ 0.012, Figure S1). A graphical abstract of the main results of the present study is depicted in Fig. 4.

## Discussion

In the present study, we show that the fecal microbiota diversity and composition are positively correlated with the plasma levels of the secondary BA GLCA in young adults. In addition, our study reveals that the relative abundance of bacterial species belonging to the *Firmicutes* and *Bacteroidetes* phyla is negatively correlated with the plasma levels of primary BA (i.e., CA, CDCA, GCA, and GCDCA) and secondary BA (i.e., DCA, GDCA, and GLCA) (Fig. 4). These findings suggest that the relative abundance of specific gut bacterial species might modulate the plasma levels of BA in young adults.

Overall, we found no relationship between fecal microbiota diversity and plasma levels of primary and secondary BA, which concurs with the homogeneity of the phenotypic characteristics of our participants. However, we found significant differences in beta diversity between participants with low and high plasma levels of GLCA. Besides, the alpha diversity indexes were positively associated with the plasma levels of GLCA, which suggests that the relative abundance of specific genera and species might be explaining the observed differences within the high GLCA concentration tertile.

In vitro experiments have shown that bacterial species belonging to the *Firmicutes* phylum (i.e., *Clostridium leptum*) and *Bacteroidetes* phylum (i.e., *Bacteroides dorei*) express either BSHs or 7-α-dehydroxylases enzymes (Gu et al., 2017; Stellwag & Hylemon, 1979). We observed that the relative abundance of *Clostridium leptum* (*Firmicutes* phylum) was negatively correlated with plasma levels of the primary BA CA, CDCA, GCA, and GCDCA. In agreement with our findings, it has been shown that the relative abundance of *Clostridium leptum* is negatively associated with the content of primary BA CA and CDCA in feces of non-alcoholic steatohepatitis patients (Mouzaki et al., 2016). These findings suggest that *Clostridium leptum*, via 7-α-dehydroxylase activity, could be involved in the production of secondary BA.

Additionally, we observed that the relative abundance of *Bacteroides dorei* (*Bacteroidetes* phylum) was negatively correlated with the plasma levels of the primary BA GCDCA, as well as with the plasma levels of the secondary BA DCA, GDCA, and GLCA. From a clinical point of view, the *Bacteroides* genus is known to have an important role in maintaining a eubiosis status (Wexler & Goodman, 2017). Preclinical studies in diet-induced obese mice showed that the administration of *Bacteroides dorei* increases the expression of the ileal BA transporter in enterocytes (Zhang et al., 2015), which is associated with a significant decrease in body weight and an improved lipid and glucose profile via farnesoid X receptor activation (Hylemon et al., 2009; Zhang et al., 2015).

Our study shows that the relative abundance of *Clostridium leptum* (*Firmicutes* phylum) and *Bacteroides dorei* (*Bacteroidetes* phylum) species is related to the plasma levels of primary and secondary BA. However, none of the relative abundances of these bacteria was related to the BA pathways, whereas the relative abundance of other bacteria did. Thus, whether other bacteria species belonging to that same phylum could be involved in the metabolism of BA deserves further investigation.

### Clinical and metabolic relevance

Whereas the liver is the site of production of primary BA, the gut microbiota bacteria modify them to give rise to a wide range of molecules with different biological and signaling functions. Growing evidence shows that alterations in the circulating levels and/or pool of BA are linked to obesity, type 2 diabetes, metabolic (dysfunction) associated fatty liver disease (MAFLD), and even cancer (Fu et al., 2022; Molinaro et al., 2018). Hence, it is of clinical relevance to study the link between gut microbiota composition and circulating BA not only in individuals suffering from these conditions but also in healthy populations with which to have a reference range of both gut microbiota composition and circulating levels of BA.

### Limitations and strengths

This study presents some limitations and, therefore, the results should be interpreted with caution. First, the cross-sectional design does not allow for the establishment of any cause-effect direction. Moreover, our cohort was relatively homogenous in terms of age and body composition, which does not enable the extrapolation of these results to other populations differing from these phenotypic characteristics. Then, we did not measure tauro-conjugated BA. The BA biosynthesis pathways are predictions based on the relative abundance of the microbial community identified by the 16S rRNA technique which is very limited. Finally, information regarding probiotics or antibiotics intake, which are known to impact the composition of gut microbiota, was not documented. Therefore, this variable could not be accounted for in our analysesOn the other hand, the two major strengths in this study are (i) we performed the DNA sequencing with one of the latest technologies (*Illumina* platform) and using the DADA2 program; and (ii) the annotation step was conducted by RDP until the species taxon, a methodology with an annotation error less than 10% (Edgar, 2018).

## Conclusion

Our findings support the hypothesis that specific bacterial species, especially those related to the *Firmicutes* and *Bacteroides* phylum, could be modulating the plasma levels of BA in humans. Nonetheless, further research is needed to better understand whether a causal relationship exists between the bacterial species expressing enzymes involved in BA metabolism with the circulating levels of BA in humans.

## Supplementary Information

Below is the link to the electronic supplementary material.

Supplementary file1 (DOCX 362 KB)

## Data Availability

The data supporting the findings of this study are available upon reasonable request. The data will be made available in compliance with applicable ethical and legal requirements.
