# Supplementary material for: The relative abundance of fecal bacterial species belonging to the Firmicutes and Bacteroidetes phyla is related to plasma levels of bile acids in young adults
Source: Metabolomics. 2023 Jun 6;19(6):54. doi: 10.1007/s11306-023-02016-8 (PMC10244271; doi:10.1007/s11306-023-02016-8)
Supplement: Supplementary file 1 — Supplementary file1 (DOCX 362 KB) [file 11306_2023_2016_MOESM1_ESM.docx]

**SUPPLEMENTARY INFORMATION**

1. **Fecal analysis**

*Sequencing*

Extracted DNA was amplified by PCR targeting the V3 and V4 hypervariable regions of the bacterial 16S rRNA gene using the following primer pairs: 16S Amplicon PCR Forward Primer: 5´CCTACGGGNGGCWGCAG; and 16S Amplicon PCR Reverse Primer: 5′GACTACHVGGGTATCTAATCC. The PCR assays were carried out in a 25 µL final reaction volume, including 12.5 µL of the 2X KAPA HiFi Hotstart prepared mixture (KAPA Biosystems, Woburn, MA, USA), 5 µL of each forward and reverse primer (1 µM), and 2.5 µL of extracted DNA (10 ng). The following PCR program was used: (i) denaturation at 95°C for 3 min, (ii) 8 denaturation cycles at 95°C for 30 s, (iii) annealing at 55°C for 30 s, (iv) elongation at 72°C for 30 s, (v) final extension at 72°C for 5 min. Next, AMPure XP microspheres (Beckman Coulter, Indianapolis, IN, USA) were used to purify the 16S V3 and V4 amplicon away from free primers and primer-dimer species. For the PCR index step, we used the Nextera XT index kit (Illumina, San Diego, CA, USA) to tag DNA with the sequencing adapters. The pooled PCR products were purified using AMPure XP balls (Beckman Coulter, Indianapolis, IN, USA) before quantification. The amplicons were sequenced in a MiSeq (Illumina, San Diego, CA, USA), using the Illumina MiSeq paired-end sequencing system (2x300nt) (Illumina, San Diego, CA, USA).

*Bioinformatics analysis*

The *dada2* (Callahan et al. 2016) package version 1.10.1 in *R* software (R Core Team 2019) was used for analyzing the raw sequences (FastQ files). All samples that were above the 10,000 reads cut-off threshold were considered valid for subsequent analyses. Samples were standardized to an equal sequencing depth of 30,982 reads using the *phyloseq* (McMurdie and Holmes 2013) package in *R* software, leading to a total of 11,158 different phylotypes.

Phylotypes were assigned to their specific taxonomic affiliation (from phylum to genus) based on the naive Bayesian classification with a pseudo-bootstrap threshold of 80% (McMurdie and Holmes 2013) using the “*Classifier*” function in Ribosomal Data Project (RDP) (Cole et al. 2014). We obtained a total of 209 genera that belong to 16 different phyla. To determine the species taxonomies, we used the “S*eqmatch*” function in RDP. For the main analysis, we used relative abundances as the read’s percentage of each phylotype relative to the total number of reads. We performed the analyses when the average of the relative sequence abundance was higher than 1%. Only those species with ≥ 97% coincidence with the respective representative sequence read and found in at least 50% of the participants were annotated, identifying a total of 50 different species for the analyses.

Beta diversity indicates the number of species shared among the microbial community from the individuals, and was assessed by pseudo-F statistics. Pseudo-F shows the ratio between cluster variance and within-cluster variance [(between-cluster-sum-of-squares/(c-1))/(within-cluster-sum-of-squares/(n-c))], where c is the number of clusters and n is the number of variables (Anderson 2017). A pseudo-F value of 1 indicates that the variance between and within-group is similar; if pseudo-F ≥ 1, the between-group variance is higher than the within-group variance. Alpha diversity reflects the number of different phylotypes and the relative abundance of these phylotypes within the same participant. A total of 4 different alpha diversity indexes were calculated: i) *species richness* (number of different phylotypes in the community) (Kim et al. 2017a); ii) *evenness index* (equitability of the phylotypes frequencies in the community) (Lozupone and Knight 2008); iii) *Shannon index* (number and equitability of the phylotypes in the community) (Kim et al. 2017b); iv) *inverse Simpson index* (derived from the classical Simpson index; richness in a community with uniform evenness) (Simpson 1949).

We also annotated microbial genes according to the Kyoto Encyclopedia of Genes and Genomes (KEGG) orthology (KOs). The metabolic contribution was inferred from 16S rRNA gene data using the Tax4fun(Aßhauer et al. 2015) program built-in Galaxy(Afgan et al. 2018) private instance of the DengLab(Feng et al. 2017) (<http://mem.rcees.ac.cn:8080>). For the present study, we included prediction of the bile acids pathways based on the microbial community with this technique.

1. **Determination of the plasma levels of bile acids**

*Sample preparation*

The sample preparation was performed on ice, except for the evaporation step. BA were extracted using liquid-liquid extraction. Before the extraction, 150 µL of plasma sample was transferred into a 1.5 mL Eppendorf tube and mixed with 5 µL of an antioxidant solution composed of 0.4 mg/mL of butylated hydroxytoluene (BHT), and 10 µL of an internal standard solution containing the isotopically-labeled analogs (**Table S1**)(DOI: [10.6084/m9.figshare.17086064](https://doi.org/10.6084/m9.figshare.17086064)) (Xu et al. n.d.). Then, 150 µL of buffer solution (0.2 M citric acid and 0.1 M disodium hydrogen phosphate at pH 4.5) were added, followed by the addition of 1 mL extraction solvent composed of methyl-tertbutylether and butanol (50:50, *v*/*v*). Samples were then mixed for 5 min using a bullet blender (Next Advance, Averill Park, NY) and centrifugated (16,000 g, 10 min, 4 °C). Next, 900 µL of supernatant were transferred to a new 1.5 mL Eppendorf tube and evaporated to dryness using a SpeedVac system at room temperature. The dry residue was reconstituted in 50 µL of methanol:acetonitrile (70:30, *v/v*), and centrifuged (16,000 g, 10 min, 4°C). Finally, 40 µL of the supernatant was transferred into an autosampler vial and 10 µL was injected into the LC-MS/MS system.

*Liquid chromatography-tandem mass spectrometry*

LC-MS/MS analysis was performed as previously described (Di Zazzo et al. 2020). Briefly, the extracted samples were analyzed using a Shimadzu LC system (Shimadzu Corporation, Kyoto, Japan), coupled to a SCIEX QTRAP 6500+ mass spectrometer (SCIEX, Framingham, MA). The separation was carried out using a BEH C18 column (50 mm × 2.1 mm, 1.7 μm) from Waters Technologies (Milford, MA) kept at 40°C. The mobile phase consisted of 0.1% acetic acid in water (A), 0.1% acetic acid in acetonitrile/methanol (90:10, *v/v*, B) and 0.1% acetic acid in isopropanol (C). The data acquisition was performed using electrospray ionization in negative mode. MS/MS acquisition was carried out using Selected Reaction Mode (SRM). SRM transitions were individually optimized for targeted analytes and their respective internal standards using standard solutions.

*Data pre-processing*

For each target compound, the ratio between its peak area and the peak area of its respective internal standard was calculated using SCIEX OS-MQ Software and was used for further data analysis. The data quality was monitored using regular injection of quality control (QC) samples, prepared from of blank plasma samples. QC samples were used to correct for between batch variations, using the in-house developed mzQuality workflow (available at http://www.mzQuality.nl)(14)(Di Zazzo et al. 2020). Relative standard deviations (RSDs) were calculated for each analyte present in the QC samples (**Table S2**)(DOI: [10.6084/m9.figshare.17086064](https://doi.org/10.6084/m9.figshare.17086064)) (Xu et al. n.d.). All analytes showed RSD values in QC samples below 9%, ensuring high data quality.

**SUPPLEMENTARY INFORMATION REFERENCES**

Afgan, E., Baker, D., Batut, B., Van Den Beek, M., Bouvier, D., Ech, M., et al. (2018). The Galaxy platform for accessible, reproducible and collaborative biomedical analyses: 2018 update. *Nucleic Acids Research*, *46*(W1), W537–W544. https://doi.org/10.1093/nar/gky379

Anderson, M. J. (2017). Permutational Multivariate Analysis of Variance ( PERMANOVA ). In *Wiley StatsRef: Statistics Reference Online* (pp. 1–15). Wiley. https://doi.org/10.1002/9781118445112.stat07841

Aßhauer, K. P., Wemheuer, B., Daniel, R., & Meinicke, P. (2015). Tax4Fun: Predicting functional profiles from metagenomic 16S rRNA data. *Bioinformatics*, *31*(17), 2882–2884. https://doi.org/10.1093/bioinformatics/btv287

Callahan, B. J., Mcmurdie, P. J., Rosen, M. J., Han, A. W., Johnson, A. J. A., & Holmes, S. P. (2016). DADA2: High resolution sample inference from Illumina amplicon data. *Nature Methods*, *13*, 581–583. https://doi.org/10.1038/nmeth.3869

Cole, J. R., Wang, Q., Fish, J. A., Chai, B., McGarrell, D. M., Sun, Y., et al. (2014). Ribosomal Database Project: Data and tools for high throughput rRNA analysis. *Nucleic Acids Research*, *42*(D1). https://doi.org/10.1093/nar/gkt1244

Di Zazzo, A., Yang, W., Coassin, M., Micera, A., Antonini, M., Piccinni, F., et al. (2020). Signaling lipids as diagnostic biomarkers for ocular surface cicatrizing conjunctivitis. *Journal of Molecular Medicine*, *98*(5), 751–760. https://doi.org/10.1007/s00109-020-01907-w

Feng, K., Zhang, Z., Cai, W., Liu, W., Xu, M., Yin, H., et al. (2017). Biodiversity and species competition regulate the resilience of microbial biofilm community. *Molecular Ecology*, *26*(21), 6170–6182. https://doi.org/10.1111/mec.14356

Kim, B.-R., Shin, J., Guevarra, R. B., Lee, J. H., Kim, D. W., Seol, K.-H., et al. (2017a). Deciphering Diversity Indices for a Better Understanding of Microbial Communities. *J. Microbiol. Biotechnol*, *27*(12), 2089–2093. https://doi.org/10.4014/jmb.1709.09027

Kim, B.-R., Shin, J., Guevarra, R. B., Lee, J. H., Kim, D. W., Seol, K.-H., et al. (2017b). Deciphering Diversity Indices for a Better Understanding of Microbial Communities. *J. Microbiol. Biotechnol*, *27*(12), 2089–2093. https://doi.org/10.4014/jmb.1709.09027

Lozupone, C. A., & Knight, R. (2008, July). Species divergence and the measurement of microbial diversity. *FEMS Microbiology Reviews*. https://doi.org/10.1111/j.1574-6976.2008.00111.x

McMurdie, P. J., & Holmes, S. (2013). Phyloseq: An R Package for Reproducible Interactive Analysis and Graphics of Microbiome Census Data. *PLoS ONE*, *8*(4). https://doi.org/10.1371/journal.pone.0061217

R Core Team. (2019). R: A Language and Environment for Statistical Computing.

Simpson, E. H. (1949). Measurement of Diversity. *Nature*, *163*(4148), 688–688. https://doi.org/10.1038/163688a0

Van Der Kloet, F. M., Bobeldijk, I., Verheij, E. R., & Jellema, R. H. (2009). Analytical error reduction using single point calibration for accurate and precise metabolomic phenotyping. *Journal of Proteome Research*, *8*(11), 5132–5141. https://doi.org/10.1021/pr900499r

Xu, H., Osuna-Prieto, F. J., Ortiz-Alvarez, L., & Al, E. (n.d.). Relative abundance of Firmicutes and Bacteroidetes bacteria is related to plasma levels of bile acids in young adults. *Figshare*. https://doi.org/10.6084/m9.figshare.17086064

| **Table S1.** List of internal standards. |  |
| --- | --- |
| **Abbreviation** | **Name (International Union of Pure and Applied Chemistry, IUPAC)** |
| d4-CA-ISTD | 3α,7α,12α-trihydroxy-5β-cholan-24-oic acid-d4 |
| d4-GCA-ISTD | N-(3α,7α,12α-trihydroxy-5β-cholan-24-oyl)-glycine-d4 |
| d4-DCA-ISTD | 3α,12α-dihydroxy-5β-cholan-24-oic acid-d4 |
| d4-GDCA-ISTD | N-(3α,12α-dihydroxy-5β-cholan-24-oyl) glycine-d4 |
| d5-GUDCA-ISTD | N-(3α,7β-dihydroxy-5β-cholan-24-oyl)-glycine-d5 |
| CA: cholic acid; DCA: deoxycholic acid; GCA: glycocholic acid; GDCA: glycodeoxycholic acid; GUDCA: glycoursodeoxycholic acid. | |

| **Table S2.** Bile acids abbreviations and observed variability in peak area ratios in quality control samples. | | | |
| --- | --- | --- | --- |
| **Abbreviation** | **Name (International Union of Pure and Applied Chemistry, IUPAC)** | **ChEBI ID** | **RSD of QCs** |
| *Primary bile acids* | | | |
| CA | 3α,7α,12α-trihydroxy-5β-cholan-24-oic acid | 16359 | 7.1% |
| CDCA | 3α,7α-dihydroxy-5β-cholan-24-oic acid | 16755 | 7.0% |
| GCA | N-(3α,7α,12α-trihydroxy-5β-cholan-24-oyl)-glycine | 17687 | 6.6% |
| GCDCA | N-(3α,7α-dihydroxy-5β-cholan-24-oyl)-glycine | 3593 | 5.8% |
| *Secondary bile acids* | | | |
| DCA | 3α,12α-dihydroxy-5β-cholan-24-oic acid | 28834 | 8.3% |
| GDCA | N-(3α,12α-dihydroxy-5β-cholan-24-oyl) glycine | 27471 | 6.0% |
| GLCA | N-[(3α,5β)-3-hydroxy-24-oxocholan-24-yl]-glycine | 37998 | 7.4% |
| GUDCA | N-(3α,7β-dihydroxy-5β-cholan-24-oyl)-glycine | 89929 | 7.0% |
| Observed variability is expressed as relative standard deviation of the peak area ratio in the quality control samples. CA: cholic acid; CDCA: chenodeoxycholic acid; ChEBI: Chemical Entities of Biological Interest; DCA: deoxycholic acid; GCA: glycocholic acid; GCDC: glycochenodeoxycholic acid; GDCA: glycodeoxycholic acid; GLCA: glycolithocholic acid; GUDCA: glycoursodeoxycholic acid; QC: quality control; RSD: relative standard deviation. | | | |


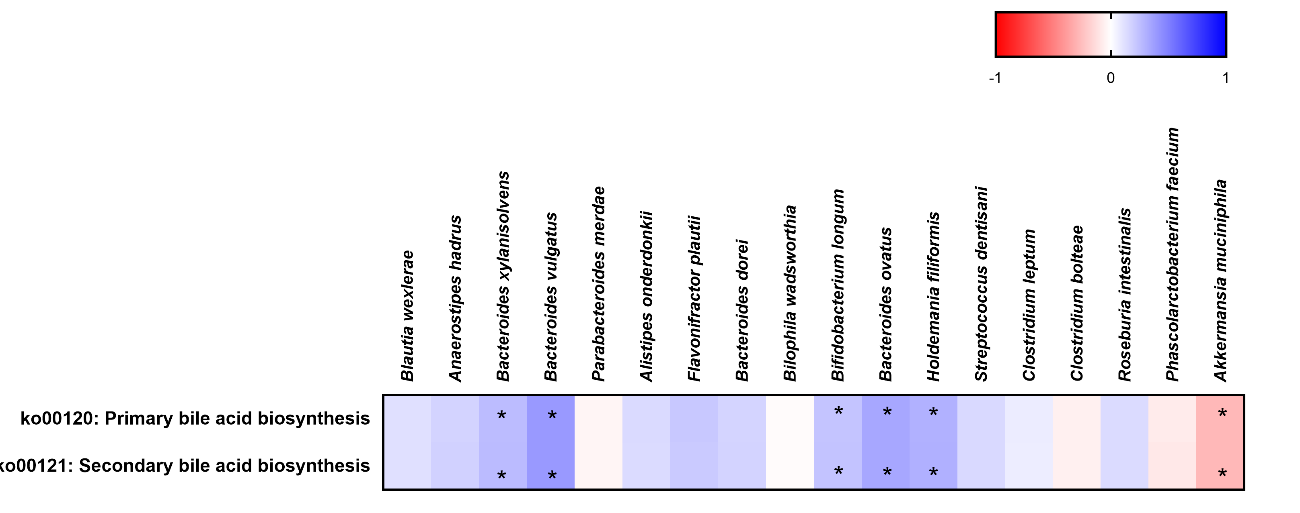


**Figure S1**: Spearman correlation between specific bacterial species and predictions of primary and secondary bile acids biosynthesis pathways. Symbols * mean significant correlation (P<0.05).
